# Evaluation of a novel permanent capped helical coil fastener in a porcine model of laparoscopic ventral hernia repair

**DOI:** 10.1007/s00464-016-4874-1

**Published:** 2016-04-08

**Authors:** Arnab Majumder, Mojtaba Fayezizadeh, William W. Hope, Yuri W. Novitsky

**Affiliations:** 1 Department of Surgery, Case Comprehensive Hernia Center, University Hospitals Case Medical Center, 11100 Euclid Avenue, Cleveland, OH 44106 USA; 2 Department of Surgery, New Hanover Regional Medical Center, Wilmington, NC USA

**Keywords:** Hernia repair, Laparoscopy, Permanent fixation, Fastener, Helical coil

## Abstract

**Background:**

Existing permanent helical coil fasteners, although commonly employed for mesh fixation during laparoscopic hernia repair, are associated with peritoneal tissue attachment formation and resultant visceral complications. We evaluated attachment formation, fastener engagement, and mesh/tissue integration associated with laparoscopic fixation using a novel permanent capped helical coil fastener (HC-Capped) compared to permanent non-capped helical coil fasteners (HC-Non-Capped) in a porcine model.

**Methods:**

Twelve female pigs underwent bilateral laparoscopic intraperitoneal fixation of Composix™ L/P Mesh (10 × 15 cm oval) with HC-Capped or HC-Non-Capped fasteners. Thirty-two fasteners were used to secure each mesh utilizing a “double-crown” technique. Laparoscopy at 30 days was used to evaluate the presence and area coverage of attachments (Diamond Score) and percentage of engaged fasteners. At 90 days, following necropsy, each mesh was evaluated for the presence, percentage, and tenacity (Butler Score) of attachments and fastener engagement. Samples were biomechanically evaluated to assess tissue integration via T-peel testing.

**Results:**

HC-Capped fasteners demonstrated a significantly lower attachment area score compared to the HC-Non-Capped group at 30 days (0.92 ± 0.26 vs. 2.50 ± 0.29/3.00, *p* = 0.002) and 90 days (0.60 ± 0.22 vs. 2.08 ± 0.29/3.00, *p* = 0.004). At 90 days, the HC-Capped group evidenced significantly lower attachment tenacity score (1.00 ± 0.37 vs. 2.75 ± 0.33/4.00, *p* = 0.013). Furthermore, at 30 and 90 days, a significantly greater percentage of HC-Capped fasteners remained properly engaged (30 days: 99.7 % vs. 86.5 %, *p* < 0.001 and 90 days: 99.4 % vs. 74.5 %, *p* = 0.001). T-peel biomechanical testing demonstrated significantly greater mesh/tissue integration for HC-Capped group (2.16 ± 0.24 vs. 1.16 ± 0.29 N/cm, *p* = 0.038).

**Conclusions:**

In a porcine model, HC-Capped fasteners demonstrated significantly less attachment coverage and tenacity in the early postoperative period. Furthermore, the HC-Capped cohort evidenced significantly greater percentage of properly engaged fasteners and greater mesh/tissue integration. Data suggest that shielding exposed fastener points on the visceral mesh surface with polymer caps may reduce attachment formation and aid in mesh fixation and integration.

Laparoscopic ventral hernia repair has evolved as an effective alternative to open repair since its introduction in 1993 by LeBlanc and Booth [1]. Advancements in technology and surgeon experience have since increased the popularity and widespread adoption of the laparoscopic approach for hernia repair. A recent meta-analysis has shown that while recurrence rates are at least equivalent to open repair, perioperative wound morbidity and length of stay are often superior for the laparoscopic approach [2, 3]. Although patient selection remains essential for successful outcomes, non-patient factors including mesh type, fixation method, and defect closure are all fundamental aspects to a robust and durable repair. Among these factors, mesh fixation during laparoscopic ventral hernia repair is integral to the success of the procedure with many commercially available options.

Laparoscopic mesh fixation as originally introduced by LeBlanc consisted of a suture to stabilize the mesh, followed by staples delivered along the periphery of the mesh for fixation [1]. Subsequent development of helical coil fasteners led to a variable adoption of tacking as a frequent and often sole method of fixation. Wassenaar et al. [4] showed that the double-crown fixation technique with the ProTack™ device (Covidien, Plc., New Haven, CT) did not lead to any increase in recurrence rates or perioperative morbidity, compared to transabdominal sutures in a series of 138 patients. With continued favorable results and steady innovation, widespread adoption of fixation devices as a sole method or combined method for fixation has been seen in recent years [5].

However, despite improved clinical efficacy and ease of use, commonly employed non-capped helical coil fasteners are not without their drawbacks. The sharp edges and exposed metal on the visceral mesh surface may predispose to peritoneal tissue attachment formation and resultant visceral complications [6–8]. To address the shortcomings of currently available permanent fixation devices, a permanent polymer cap made of polyether ether ketone (PEEK) was recently developed. The objective of this study was to evaluate peritoneal tissue attachments, fastener engagement, and mesh/tissue integration associated with laparoscopic fixation of a composite mesh using novel permanent capped helical coil fasteners (HC-Capped), compared to permanent non-capped helical coil fasteners (HC-Non-Capped) in a porcine model.

## Materials and methods

Animal protocol approval was obtained from the Institutional Animal Care and Use Committee (IACUC) at University Hospitals Case Medical Center. A total of 12 adult female Yorkshire pigs (52.7–56.9 kg) were randomized to receive mesh fixation via a novel capped helical coil fasteners (HC-Capped) or existing non-capped helical coil fasteners (HC-Non-Capped). All animals then underwent bilateral laparoscopic implantation of 10 × 15 cm oval Composix™ L/P Mesh (C.R. Bard, Inc. (Davol), Warwick, RI) as an underlay (without defect creation) with one device type used per animal (*n* = 6 pigs, *n* = 12 mesh/group). A total of 32 fasteners were used to secure each mesh utilizing a “double-crown” technique (*n* = 384 fasteners deployed/group). The animals were survived for a total of 90 days under standard conditions, with daily monitoring and access to food, water, and enrichment, ad libitum.

### Fixation devices

The two devices that were compared in this study are as follows: HC-Capped test device (CapSure™, C. R. Bard, Inc., Warwick, RI) and HC-noncapped control device (ProTack™, Covidien, Plc., New Haven, CT). The HC-Capped permanent fasteners are made from a 316L stainless steel with a helical coil integrated to a fastener head made from polyether ether ketone (PEEK). The fasteners are 4.2 mm in length with a total of 30 fasteners per device. Comparatively, the HC-Non-Capped permanent fasteners are helical in shape and made from titanium. The fasteners are 3.8 mm in length with a total of 30 fasteners per device. Both devices have a shaft with an outer diameter of 5 mm for use in open procedures or with most 5-mm trocars in laparoscopic procedures.

### Surgery

**Fig. 1:**
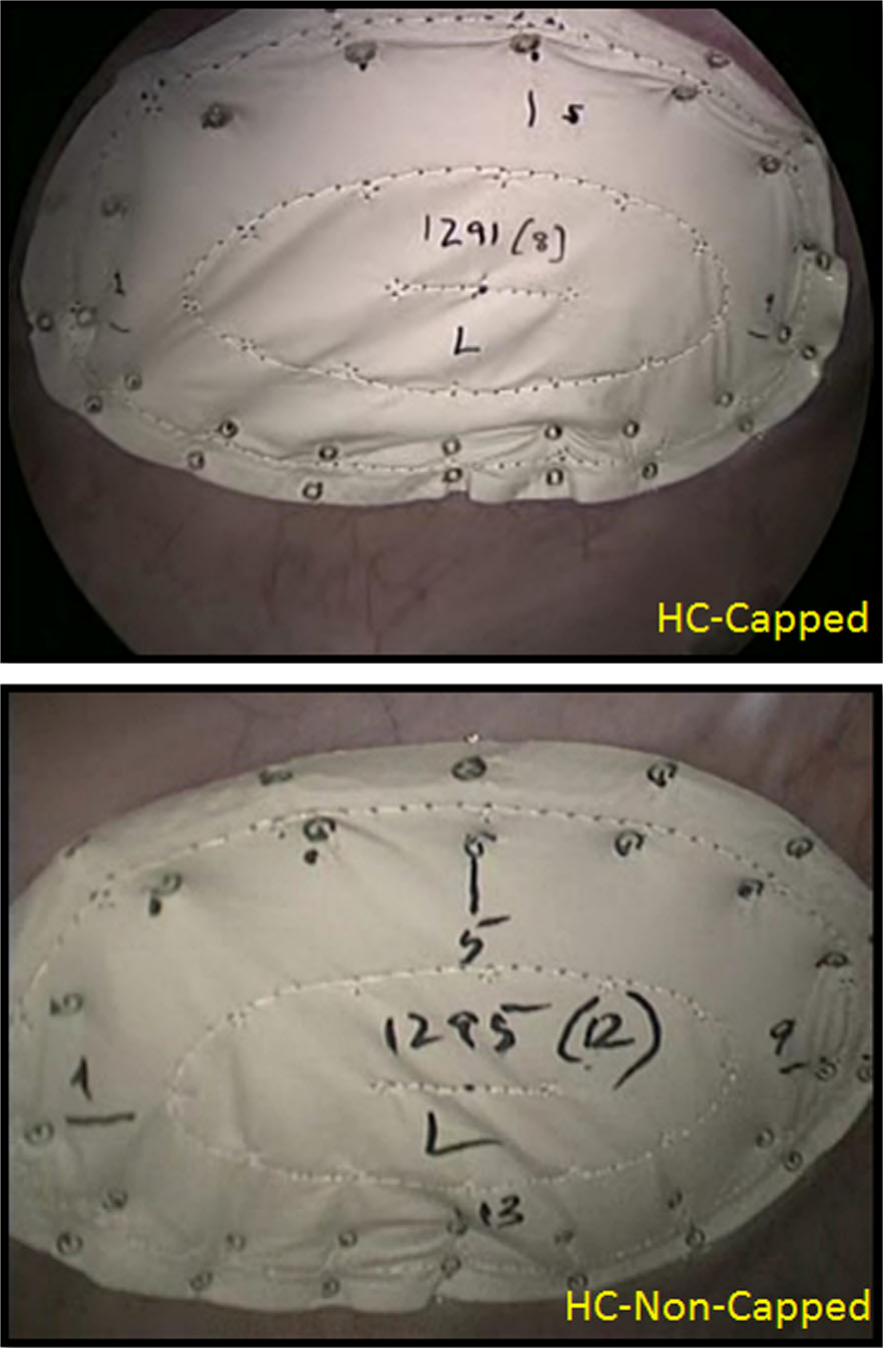
Implantation photographs: Intraperitoneal fixation of Composix™ L/P Mesh (10 × 15 cm oval) with HC-Capped or HC-Non-Capped fasteners. Thirty-two (*n* = 32) fasteners were used to secure each mesh utilizing a “double-crown” technique

After intramuscular induction of anesthesia with Telazol^®^ 8 mg/kg (Zoetis Inc., Kalamazoo, MI), each animal underwent endotracheal intubation and was placed supine on an operating table. General anesthesia was maintained with inhaled isoflurane, with continuous monitoring of vital signs including pulse oximetry. Excess hair was trimmed and the abdomen was widely prepped with alcohol and chlorhexidine solution. The animal was then draped in standard fashion utilizing Ioban™ (3 M, St. Paul, MN). Analgesia was provided with a preoperative dose of intramuscular buprenorphine (0.05 mg/kg), and local anesthesia was administered using a lidocaine (2 mg/kg) and bupivacaine (4 mg/kg) mixture. A Hasson cut-down technique was performed in the subxiphoid region, with subsequent insertion of a 10-mm trocar. The abdominal cavity was inspected to facilitate safe trocar placement, and two 5-mm ports were placed laterally away from the midline on the right and left sides of the animal (under direct vision). Mesh was rolled then introduced through the 10-mm port. Each mesh was secured against the abdominal wall via a central stay suture snared by a straight suture passer. Once in position, the mesh was secured to the abdominal wall lateral to the midline on each side utilizing a double-crown method (32 fasteners per mesh) with even spacing at pre-marked locations (Fig. 1). The abdomen was inspected again to ensure that both meshes were secured to the abdominal wall. Trocars were removed, and pneumoperitoneum was released. Fascia at the cut-down site was closed with a running 2-0 Polysorb™ suture (Covidien, Plc., Minneapolis, MN), and subcutaneous tissue was re-approximated using 3-0 Polysorb™ suture. Skin was closed using a running 4-0 Caprosyn™ suture (Covidien, Plc., Minneapolis, MN). Lateral port sites were closed with interrupted 3-0 Polysorb™ for deep tissue and 4-0 Caprosyn™ for the skin. Animals were recovered from anesthesia under direct observation, with monitoring of vital signs every 15 min for the first hour, and every 30 min until the animal was sternal. Postoperative pain control was provided with oral Carprofen (4 mg/kg) once daily for 3 days, along with a Fentanyl patch (25 mcg/h) for 72 h. Daily assessments were made of each animal throughout the survival period.

**Fig. 2:**
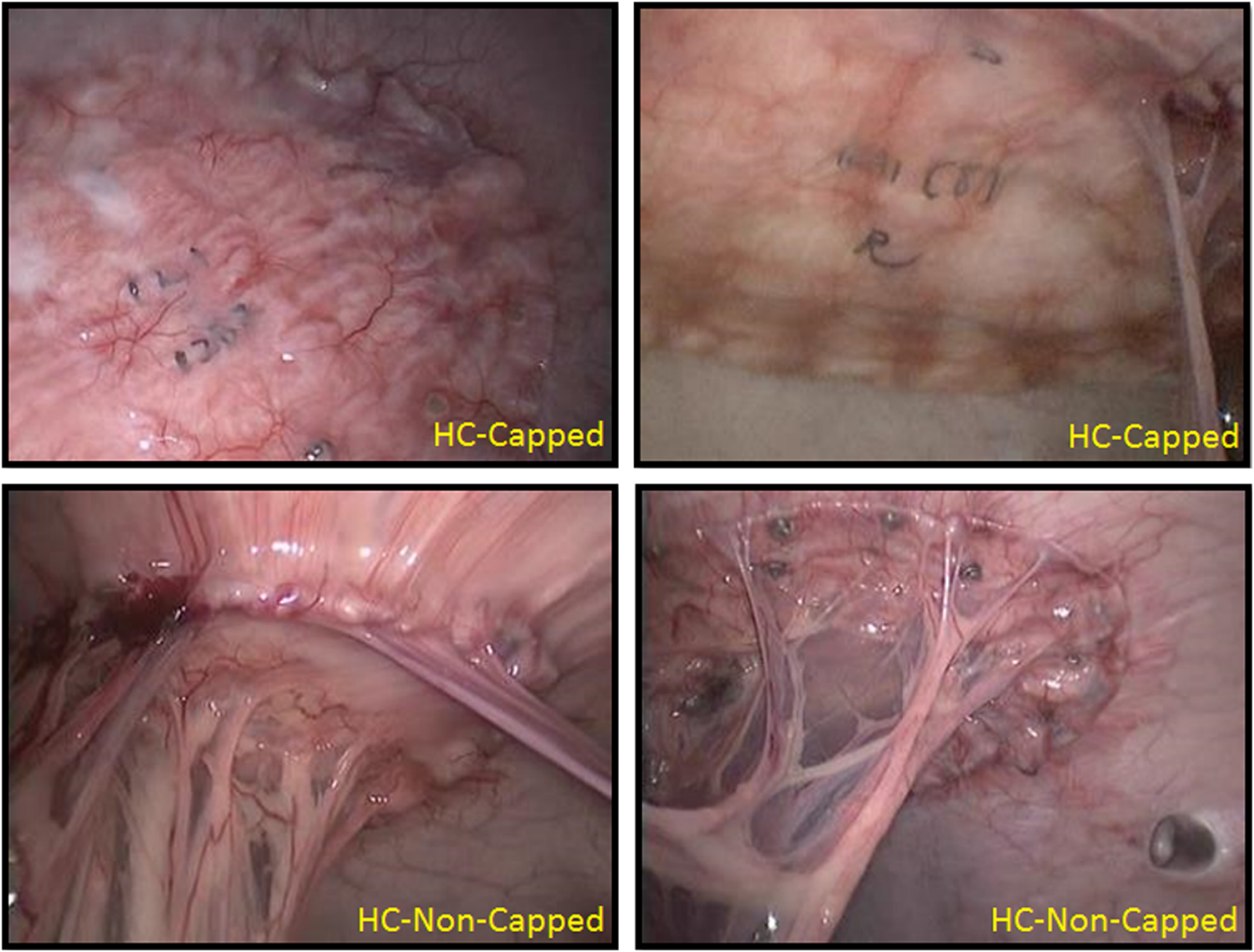
30-day laparoscopic fastener assessment photographs: at 30 days post-implantation, all animals underwent diagnostic laparoscopy to evaluate the presence and percent area coverage of tissue attachments, as well as proper fastener engagement. At this time point, all meshes remained secured to the abdominal wall. HC-Capped fasteners demonstrated reperitonealization and minor tissue attachments, while HC-Non-Capped fasteners demonstrated extensive tissue attachments

**Fig. 3:**
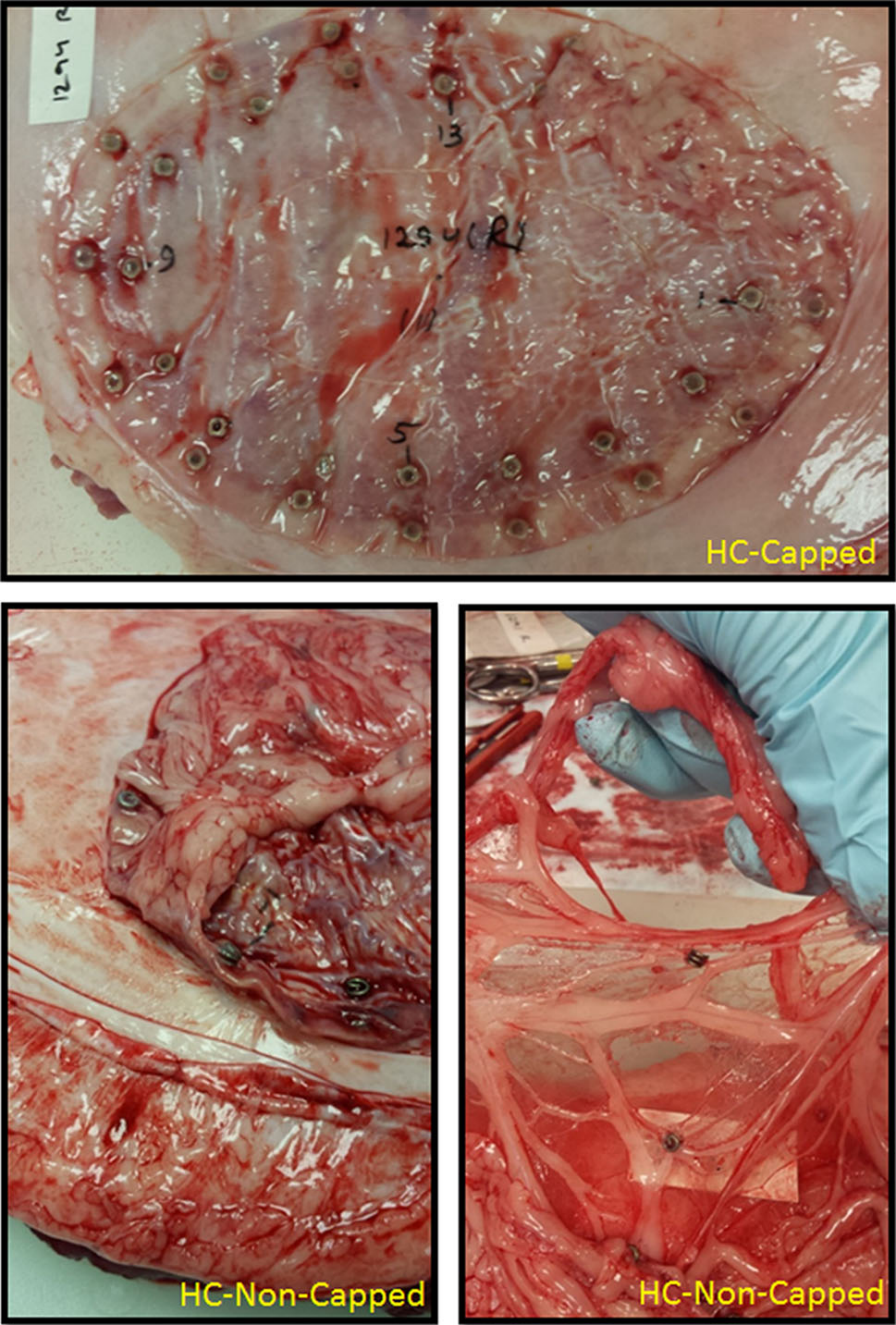
90-day gross necropsy fastener assessment photographs: at 90 days post-implantation, all meshes remained secured to the abdominal wall. Similar to the 30-day results, HC-Capped fasteners demonstrated minor tissue attachments, while HC-Non-Capped fasteners demonstrated extensive tissue attachments

At 30 days, following the same perioperative steps, all animals underwent a diagnostic laparoscopy to evaluate presence and percentage of tissue attachments, as well as proper fastener engagement (Fig. 2). Animals were given the same postoperative analgesia and survived for another 60 days. At 90 days following implantation, animals underwent euthanasia and necropsy. Animals were euthanized via an overdose (>100 mg/kg) of Fatal-Plus^®^ (Vortech Pharmaceuticals, Dearborn, MI). The abdominal wall complex was harvested in total using sharp dissection to remove the outer skin and subcutaneous tissue (down to the level of the fascia). The mesh/peritoneum complex was kept intact as a large rectangular block of tissue. Tissue attachments to the mesh/fasteners were kept intact and sharp dissection to free any attachments from the viscera and omentum was done as close to the originating point as possible. Each fixated mesh was then evaluated for presence, percent area coverage, and tenacity of tissue attachments, along with evaluation of proper fastener engagement (Fig. 3). Proper fastener engagement was defined as the fastener head being flush and in contact with surface of the mesh and tissue. Improper engagement was considered if there was a gap between the bottom of the fastener head and the surface of the mesh or if the fastener had completely backed out and was not engaged with mesh or tissue. Following gross evaluation, specimens of each fixated mesh were also mechanically evaluated to assess tissue.

### Tissue attachment evaluation

Tissue attachment area was quantified using the Diamond Tissue Attachment Scoring Scale (Table 1). During 30-day diagnostic laparoscopy and at necropsy, proper fastener engagement was evaluated. At necropsy, tissue attachment tenacity was also evaluated using the Modified Butler Tissue Attachment Tenacity Grading Scale (Table 2).

**Table 1:** Diamond Tissue Attachment Scoring Scale

Diamond Score	Percentage coverage of fasteners
0	0
1	1–25
2	26–50
3	>50

**Table 2:** Modified Butler Tissue Attachment Tenacity Grading Scale

Butler Grading Score	Tenacity of attachments
0	No attachments
1	Freed with gentle tension
2	Requiring blunt dissection
3	Requiring sharp dissection
4	Visceral organ attachment

### Biomechanical testing

Following tissue attachment evaluation, each abdominal wall/mesh sample underwent biomechanical testing by Altran Solutions (Boston, MA) to assess mesh–tissue integration.

Sample preparation: T-peel mechanical testing was conducted on a representative (2 × 6 cm) dissected product strip (without fasteners) following explanation, to quantify the degree of tissue ingrowth into the surface of the mesh devices. T-peel samples were die-cut using an arbor press and custom cutters. The sample cutters were fabricated by securing four rectangular-shaped blades into a stiff backing plate. The arbor press was used to apply pressure to the cutter, against the abdominal wall tissue with the mesh fixated, thus creating a standardized representative (2 × 6 cm) sample strip. The T-peel sample cutter was positioned to ensure that the samples were located inbound from fastener locations, so as to assess the mesh/tissue interface directly. Additionally, the sample strips were prepared for T-peel mechanical testing by dissecting a 1-cm tab to separate the mesh device from the abdominal wall peritoneum at one end across the width. The separation was made in order to allow adequate insertion of the mesh device into one pneumatic grip and the abdominal wall (peritoneum) into the other pneumatic grip. Two tensile pneumatic grips were used to hold the sample. The lower grip and the upper grip faces were standard serrated to hold the mesh device and abdominal wall peritoneum, respectively. Grip pressure was adjusted to 45 psi to limit sample slippage and damage.

To facilitate mechanical testing, a calibrated Instron^®^ (Norwood, MA) electromechanical test frame with digital data acquisition was utilized. T-peel separation was performed under a controlled displacement rate of 25 mm/min. The load and displacement information during testing was captured at 10 Hz. As the test progressed, the samples were continuously monitored noting the quality and location of the abdominal wall (peritoneum) and mesh device separation. This particular consideration was performed to make certain that the T-peel test occurred between the mesh device and abdominal wall (peritoneum), ensuring that minimal tissue/abdominal wall peritoneum remained attached to the mesh device post-separation. The samples were continuously monitored during testing, observing the quality and location of the abdominal wall–mesh device separation.

Data were recorded using the Instron^®^ digital recorder through Instron^®^ BlueHill^®^ 3 software and was processed using custom developed MS Excel^®^ 14.0 spreadsheets. Raw data were used to calculate the T-peel force by capturing the averaging force within a consistent separation range. The T-peel force was also normalized to the width of the tested specimen by dividing by the sample width (2 cm).

### Statistical analysis

Results are presented as mean ± SEM (standard error of the mean), and values were statistically compared using Wilcoxon rank-sum test (for tissue attachment scoring), Fisher’s exact test (for fastener engagement), and Student’s *t*-test (for biomechanical data).

## Results

All pigs survived the initial mesh implantation surgery and 30-day diagnostic laparoscopy procedure. There was one anesthesia-related, non-device-related death that occurred within the HC-Capped group immediately after the 30-day laparoscopy.

**Fig. 4:**
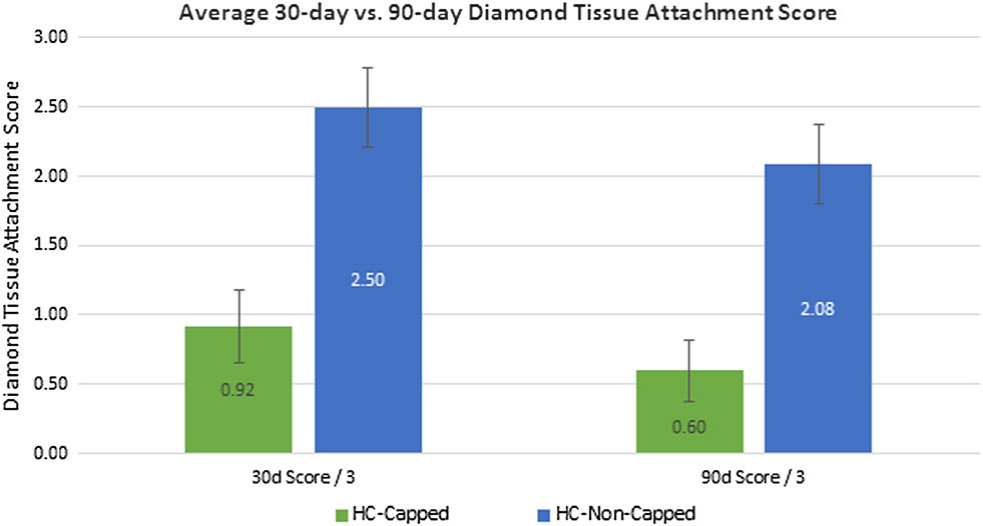
30-day versus 90-day peritoneal tissue attachment coverage score: at 30 days post-implantation, HC-Capped fasteners demonstrated rapid reperitonealization, resulting in a significantly lower intraperitoneal Diamond Tissue Attachment Score (0.92 ± 0.26/3.00) compared to HC-Non-Capped fasteners (2.50 ± 0.29/3.00), *p* = 0.002. At 90 days post-implantation, HC-Capped fasteners also demonstrated significantly lower intraperitoneal Diamond Tissue Attachment Score (0.60 ± 0.22/3.00) compared to HC-Non-Capped fasteners (2.08 ± 0.29/3.00), *p* = 0.004

**Fig. 5:**
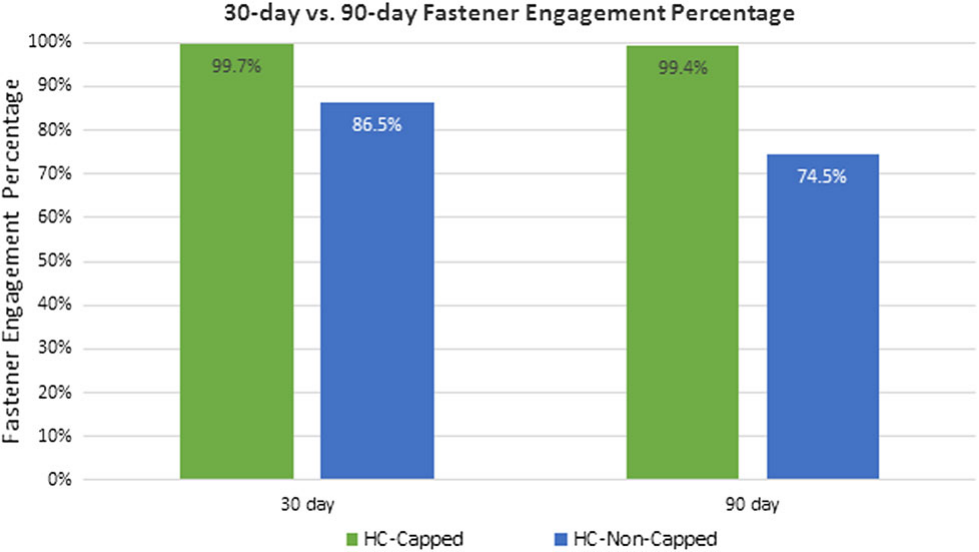
30-day versus 90-day fastener engagement percentage: at 30 days post-implantation, a significantly higher percentage of properly engaged fasteners was demonstrated for the HC-Capped group (383/384, 99.7 %) compared to the HC-Non-Capped group (332/384, 86.5 %), *p* < 0.001. Similarly, at 90 days post-implantation, a significantly higher percentage of properly engaged fasteners was demonstrated for the HC-Capped group (318/320, 99.4 %) compared to the HC-Non-Capped group (286/384, 74.5 %), *p* = 0.001

At 30 days post-implantation, all meshes remained secured to the abdominal wall. HC-Capped fasteners demonstrated rapid reperitonealization, resulting in a significantly lower intraperitoneal Diamond Tissue Attachment Score (0.92 ± 0.26/3.00), as compared to HC-Non-Capped fasteners (2.50 ± 0.29/3.00), *p* = 0.002 (Fig. 4). Additionally a significantly higher percentage of properly engaged fasteners was demonstrated for the HC-Capped group (383/384, 99.7 %), as compared to the HC-Non-Capped group (332/384, 86.5 %), *p* < 0.001 (Fig. 5).

**Fig. 6:**
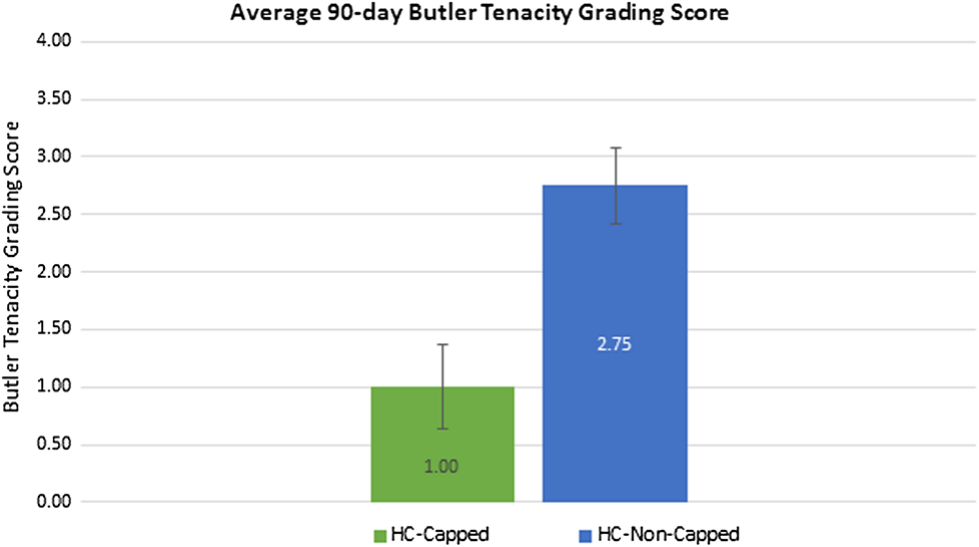
90-day peritoneal tissue attachment tenacity score: at 90 days post-implantation, the HC-Capped fasteners demonstrated a significantly lower Butler Tissue Attachment Tenacity Score (1.00 ± 0.37/4.00) compared to HC-Non-Capped fasteners (2.75 ± 0.33/4.00), *p* = 0.013

At 90 days post-implantation, all meshes again remained secured to the abdominal wall. Similar to the 30-day results, HC-Capped fasteners demonstrated a significantly lower intraperitoneal tissue attachment score (0.60 ± 0.22/3.00), as compared to HC-Non-Capped fasteners (2.08 ± 0.29/3.00), *p* = 0.004 (Fig. 4). Both groups demonstrated a nonsignificant trend toward fewer tissue attachments at 90 versus 30 days. The HC-Capped fasteners demonstrated significantly lower Butler Tissue Attachment Tenacity Score (1.00 ± 0.37/4.00), as compared to HC-Non-Capped fasteners (2.75 ± 0.33/4.00), *p* = 0.013 (Fig. 6). Again, similar to 30-day data, a significantly higher percentage of HC-Capped fasteners remained properly engaged (318/320, 99.4 %), as compared to the HC-Non-Capped group (286/384, 74.5 %), *p* = 0.001 (Fig. 5).

**Fig. 7:**
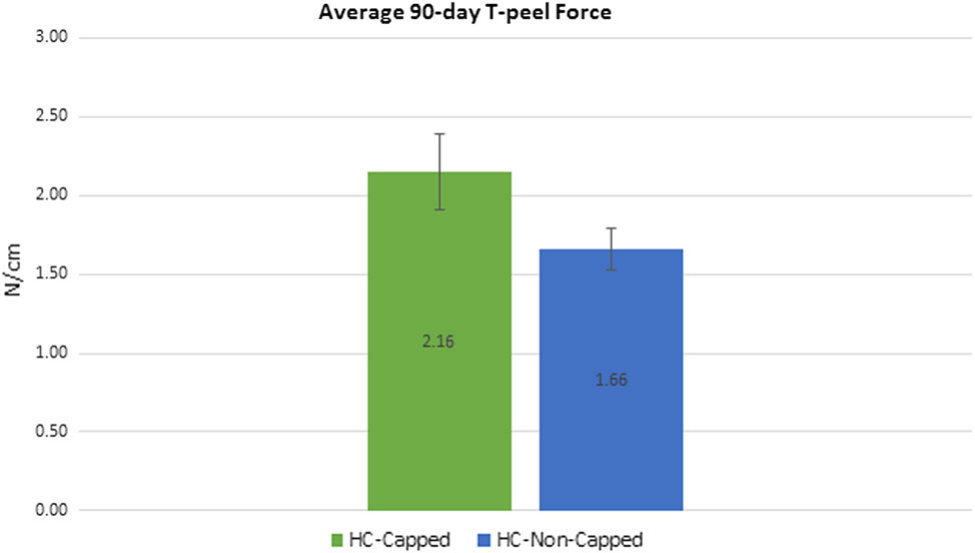
90-day tensiometric analysis: biomechanical testing revealed significantly greater tissue ingrowth via greater T-Peel force measured for meshes fixated with HC-Capped fasteners compared to HC-Non-Capped fasteners (*p* = 0.038)

Biomechanical testing revealed significantly greater tissue ingrowth via higher T-Peel force for mesh fixated with HC-Capped fasteners (2.16 N/cm ± 0.24), as compared to HC-Non-Capped fasteners (1.16 ± 0.13), *p* = 0.038 (Fig. 7). These findings are summarized in Table 3.

**Table 3:** Summary of 30-day and 90-day findings

	30-day attachment area score	90-day attachment area score	30-day engaged fastener (%)	90-day engaged fastener (%)	90-day attachment tenacity score	90-day T-peel force (N/m)
HC-Capped	0.92 ± 0.26	0.60 ± 0.22	99.7 % (383/384)	99.4 % (318/320)	1.00 ± 0.37	2.16 ± 0.24
HC-Non-Capped	2.50 ± 0.29	2.08 ± 0.29	86.5 % (332/384)	74.5 % (286/384)	2.75 ± 0.33	1.16 ± 0.13
*p* value	0.002	0.004	<0.001	0.001	0.013	0.038

## Discussion

Mesh fixation during laparoscopic hernia repair is critical to the success and durability of each procedure. Although a variety of mesh types are utilized in clinical practice, a reliable and functional method for fixation remains to be of paramount importance. Proper fixation methods should secure the mesh to the abdominal wall, with minimal associated tissue attachment formation. The use of trans-abdominal suture fixation remains debatable; however, the use of tackers remains common [5]. Although absorbable fixation methods have gained traction, permanent fixation remains a staple in the armamentarium of the laparoscopic hernia surgeon. The objective of this study was to evaluate peritoneal tissue attachments, fastener engagement, and mesh/tissue integration associated with laparoscopic fixation of a composite mesh using novel HC-Capped, compared to HC-Non-Capped fasteners in a porcine model.

Morbidity of existing non-capped titanium helical coil fasteners is well-known. The use of HC-Non-Capped fasteners has been reported to be associated with significant peritoneal tissue attachment formation both in animal models and in the clinical realm [6–8]. Although generally considered bio-inert, Byrd et al. [9] demonstrated more frequent tissue attachment formation and significantly more tenacious tissue attachments when comparing permanent titanium fasteners to permanent polymeric fasteners. Postsurgical tissue attachment formation has long been implicated as a contributing cause of postoperative visceral complications such as bowel obstruction, following abdominal surgical procedures, open or laparoscopic. The goal remains to reduce the formation of these attachments following surgery. Multiple permanent and absorbable barriers applied to mesh have been employed to address the issue of attachment formation with varying degrees of success [10, 11]. Ultimately, however, these barriers do not address the predisposition of attachment formation to the fasteners themselves [12, 13]. Following mesh fixation, fasteners remain exposed to the peritoneal cavity after driving through the mesh and protective layers, thus providing numerous foci for attachment formation. Theoretically, shielding the exposed metal surfaces should decrease the amount of tissue attachments formed and subsequently reduce the rate of visceral complications arising from attachment formation.

A permanent fastener component has long been sought as a possible solution to minimize complications related to the exposed edges of the fastener. PEEK is a semicrystalline thermoplastic that has been utilized in numerous engineering applications given its mechanical strength and chemical durability. In addition to the characteristics that make PEEK useful in industrial applications, its biocompatibility profile has seen the widespread utilization in the orthopedic realm, with direct clinical applications in spinal surgery as well as ligament repair [14–16]. Specifically, in regard to hernia repair, during the deployment of fasteners, considerable torque can be applied on the head of the fastener by the inner cannula. With precedent as material chosen for orthopedic screws and spinal cages, PEEK was utilized given its low Young’s modulus which provides for adequate torsional resilience. Furthermore, PEEK has been shown to have a minimal immunogenic response even when used in demanding spinal applications, with both in vitro and in vivo studies showing no cytotoxicity and blunted inflammatory reaction [16–18]. These characteristics make PEEK a biologically inert material and thus less likely to contribute to the development of inflammation-induced tissue attachment. Ultimately, both mechanical and biologic properties of PEEK make it an optimal substrate to tolerate the physical demands of deployment, while serving a role as a biologically inert shield for underlying metal fasteners.

Reduction in tissue attachment formation was the primary goal of shielding the exposed metal of helical coil fasteners. Prior work with titanium fasteners in murine models have shown a propensity for tissue attachment formation to these un-shielded fasteners compared to suture fixation [19]. Our results in a porcine model show that the HC-Capped group had significant reductions in not only tissue attachment formation, but the tenacity of these attachments both at 30 and 90 days. We found that HC-Non-Capped fasteners were associated with over threefold increase in tissue attachment area compared to the HC-Capped group at 90 days. Furthermore, similar to the reduction in mean tissue attachment area seen in the postoperative period, the tenacity of attachments in the HC-Capped group was also nearly threefold lower. Although a complete absence of tissue attachment formation was not seen for the HC-Capped fasteners, the average attachment observed was able to be freed with gentle tension alone, while those that formed to the control fastener were much more robust, often requiring extended dissection to free. It was evident that shielding of metal fasteners with PEEK within this study, not only reduced the rate of attachment formation, but also altered the tenacity of the attachment itself.

In addition to the iatrogenic complications caused from peritoneal tissue attachment formation to HC-Non-Capped fasteners, there are reports of significant sequelae from disengagement of these fasteners from the abdominal wall including small bowel obstruction, volvulus, and small bowel perforation [20–23]. A lack of proper fastener engagement over time can be theorized as a potential mechanism by which fasteners possibly contribute to tissue attachment formation. Primarily, fastener engagement is a function of fastener design, the way in which the device is fired, how the fasteners initially engage with the mesh/tissue, and the stability of that engagement over time. It is beyond the scope of this study to determine the difference in device characteristics and their role in fastener engagement. However, tissue attachment formation plays a role in exerting traction on the deployed fasteners, and force can then back out the fasteners on the deployment axis. Compared to screws which often utilize a beveled thread to resist physical forces and provide a secure interface, helical fasteners are not beveled but rather are cylindrical coils. Consequently, coils compared to screws are less able to resist traction and are thus more likely to become disengaged when traction is applied. However, coils connected to a proximal retention feature such as the polymer cap may allow for a tighter compression of mesh and tissue at the interface of cap and coil, theoretically facilitating tighter approximation through dynamic tissue motion. Increasing the fastener surface area by integrating a low-profile cap, may also augment the depth of penetration and facilitate more uniform fastener mesh/tissue contact and reperitonealization.

Furthermore, a reduction in tissue attachment formation and tenacity reduces the likelihood of adequate traction being applied to disengage a fastener. Our data show that when compared to HC-Non-Capped fasteners, the HC-Capped fasteners showed a 13 % comparative improvement in fastener engagement percentage at 30 days and 22 % improvement at 90 days. Fastener migration was evidenced in the HC-Non-capped group with fasteners found floating mainly in the omentum, but also near distant visceral organs. We believe that in addition to the device characteristics, the reduction in attachment formation due to shielding may have improved the ability of fasteners to remain properly engaged long after deployment.

Although reduction in tissue attachments is a crucial feature of this novel device, reliable mesh fixation is similarly important. We theorized that a more secure mesh to abdominal wall interface would result in improved ingrowth. For the HC-Capped fixated mesh we observed that nearly all (>99 %) of the deployed fasteners remained properly engaged at the completion of the experiment. Conversely, in the HC-Non-Capped group, we noted many migrated and displaced fasteners. Such inconsistency, in turn, led to the edges of the mesh curling away from the abdominal wall in the HC-Non-Capped group, resulting in poorer contact (Fig. 3). Mirroring our gross findings, biomechanical results showed nearly a doubling of the T-peel force needed to separate the mesh form the abdominal wall in the HC-Capped group. Improvement in mesh securement and interface has multiple favorable clinical implications.

Limitations of this study are inherent to any preclinical experiment. Utilization of a porcine model is widely accepted for evaluation of many novel devices and biomaterials; however, the reactivity of the material may not be representative in a human application. Furthermore, longer implantation time points may have yielded more definitive results. However, we believe the 90-day duration of the study allowed us to test our hypothesis and demonstrate potential benefits of a novel permanent HC-Capped fastener design. Nevertheless, to address those potential drawbacks of this experiment, a larger and longer study is currently being contemplated and human data are needed.

Composite mesh fixated with novel permanent HC-Capped versus HC-Non-Capped fasteners demonstrated significantly less peritoneal tissue attachment coverage and tenacity in the early postoperative period in a porcine model. Furthermore, the HC-Capped group demonstrated a significantly greater percentage of properly engaged fasteners and greater mesh–tissue integration, compared to the HC-Non-Capped group. These data suggest that shielding sharp permanent fastener points on the visceral mesh surface with a capped fastener design may reduce peritoneal tissue attachment formation and aid in securely fastening mesh to the abdominal wall. Further elucidation of the relevance of these observations will require clinical evaluation.
